# Distinct metabolic features in the plasma of patients with silicosis and dust-exposed workers in China: a case–control study

**DOI:** 10.1186/s12890-021-01462-1

**Published:** 2021-03-17

**Authors:** Changjiang Xue, Na Wu, Yali Fan, Jing Ma, Qiao Ye

**Affiliations:** Department of Occupational Medicine and Toxicology, Clinical Centre for Interstitial Lung Diseases, Beijing Chao-Yang Hospital, Capital Medical University, No. 8 Workers’ Stadium South Road, Chao-Yang District, Beijing, 100020 China

**Keywords:** Silicosis, Plasma, Metabolomics, Pulmonary function, l-arginine, Kynurenine

## Abstract

**Background:**

Silicosis is a progressive pneumoconiosis characterized by interstitial fibrosis following exposure to silica dust. The role of metabolic dysregulation in the pathogenesis of silicosis has not been investigated in detail. This study aimed to identify different metabolic features in the plasma of patients with silicosis and dust-exposed workers without silicosis in metabolomics studies.

**Methods:**

Patients with silicosis, dust-exposed workers (DEWs) without silicosis and age-matched healthy controls were recruited in a case–control study. The metabolomics analyses by ultra-high performance liquid chromatography-mass spectrometry were conducted. Distinct metabolic features (DMFs) were identified in the pilot study and were validated in the validation study. The enriched signalling pathways of these DMFs were determined. The ability of DMFs to discriminate among the groups was analysed through receiver operating characteristic (ROC) curves. The correlations between DMFs and clinical features were also explored.

**Results:**

Twenty-nine DMFs and 9 DMFs were detected and had the same trend in the pilot study and the validation study in the plasma of the DEW and silicosis groups, respectively. Sphingolipid metabolism was the major metabolic pathway in the DEWs, and arginine and proline metabolism was associated with silicosis. Twenty DMFs in the DEWs and 3 DMFs in the patients with silicosis showed a discriminatory ability with ROC curve analysis. The abundance of kynurenine was higher in Stage III silicosis than in Stage I or Stage II silicosis. l-arginine and kynurenine were both negatively correlated with the percentage of forced vital capacity predicted in silicosis.

**Conclusions:**

Distinct metabolic features in the plasma of DEWs and the patients with silicosis were found to be different. Sphingolipid metabolism and arginine and proline metabolism were identified as the major metabolic pathway in the DEW and silicosis groups, respectively. l-arginine and kynurenine were correlated with the severity of silicosis.

**Supplementary Information:**

The online version contains supplementary material available at 10.1186/s12890-021-01462-1.

## Background

Silicosis is a progressive pneumoconiosis characterized by nodular interstitial fibrosis following exposure to silica dust [1]. Disease progression of silicosis usually leads to irreversible complications and death. As observed in a recent American Thoracic Society review, workplace exposures contribute substantially to the burden of chronic respiratory diseases such as silicosis [2]. China, where industries with silica exposure have not been strongly regulated and poor personal protection due to insufficient attention, has exhibited a sustained epidemic of silicosis [3]. According to a report from China’s Ministry of Health, more than 23 million workers are exposed to crystalline silica in China [4]. Currently, the clinical diagnosis and monitoring of silicosis mainly relies on a history of occupational exposure and radiological abnormalities [5].

Crystalline silica entering the airways is engulfed by macrophages, causing necrosis of the phagocytes, and then, the internalized silica is released again and engulfed by other macrophages. The repeated process of phagocytosis, necrosis and rephagocytosis of the cells induces inflammation and activation of the reactive oxygen species system, which is associated with pulmonary interstitial fibrosis [6–8]. Proteomic analysis has suggested that some proteins have been found to be closely related to the occurrence and development of silicosis [9, 10]. Most proteins were enriched in immune system processes, oxygen transporter activity, phagosome, lysosome and extracellular matrix (ECM)-receptor interactions [11]. Our previous study showed that serum Krebs von den Lungen 6, surfactant protein D and matrix metalloproteinase-2 were potential biomarkers for diagnosing and monitoring silicosis [12]. Furthermore, many metabolites are involved in the pathogenesis of silicosis and may play a predictive role in the diagnosis and severity of the disease. Research has also suggested that cholesterol oxidation can be used as an important marker and lipid metabolism is affected and oxidative lipid damage is triggered in silicosis [13].

Metabolomics, which is a systematic investigation of all metabolic responses in a biological system, provides a comprehensive and quantitative method of studying a complete set of intracellular and extracellular metabolites [14, 15]. Metabolomics studies have helped elucidate the pathogenesis and identify potential biomarkers for the diagnosis or prognosis of pulmonary fibrosis [16, 17]. Metabolic pathway changes in sphingolipid metabolism, the arginine pathway, glycolysis, mitochondrial beta-oxidation and the tricarboxylic acid cycle were found in the lung tissues of patients with idiopathic pulmonary fibrosis (IPF) in a metabolomics study [18]. Silicosis probably has similar pathogenesis as IPF, which may be related to the pathological manifestation of the production of collagen fibres and pulmonary interstitial fibrosis [19]. The role of metabolic dysregulation in the pathogenesis of silicosis has not been investigated in detail.

To identify the distinct metabolic features (DMFs) in silicosis, we evaluated the metabolic profiles in the plasma of patients with silicosis compared with dust-exposed workers without silicosis and healthy controls using pilot and validation metabolomics studies.

## Methods

### Study design and population

This case–control study adopted a cross-sectional design and recruited a total of 170 individuals. In the pilot phase, the individuals studied were 30 patients with silicosis, 30 dust-exposed workers (DEWs) without silicosis and 20 age-matched healthy controls. There were 30 individuals in each group in the validation phase. No individual participated in both the pilot and validation studies. All patients with silicosis were sequentially obtained from the Department of Occupational Medicine and Toxicology, Beijing Chao-Yang Hospital, during a more than 2-year period (January 2018 to December 2019). They were diagnosed according to the radiological criteria of pneumoconiosis based on the 2011 International Labour Organization (ILO) classification [20]. Patients with chronic obstructive pulmonary disease, asthma, tuberculosis, autoimmune disease, uncontrolled hypertension and diabetes, severe liver and kidney dysfunction, malignant tumours, and patients who abuse alcohol were excluded. The DEWs had occupational exposure to silica dust and underwent all examinations without showing evidence of silicosis. The controls comprised 50 age-matched healthy volunteers from the health examination centre of Beijing Chao-yang Hospital during the same period of time.

Clinical data were retrieved from medical reports and included age, sex, height, weight, smoking status, occupational history, current and past medical history and family history. The occupational history (including type of exposure and start and end dates of employment) was collected, and all jobs within the working life were taken into account. The subjects with silicosis and the DEWs enrolled in the study were local residents who had been exposed to silica dust during excavation and digging (83, 69.2%), polishing and buffing (16, 13.3%), handling raw materials (14, 11.7%) and rock blasting and sand blasting (7, 5.8%). Our hospital is a regional medical centre for occupational diseases. The smoking status of all individuals was carefully determined and categorized as non-smokers, ex-smokers (had quit smoking ≥ 12 months previously), and smokers (currently smoking or had quit smoking < 12 months previously). All individuals underwent chest X-ray/chest high-resolution computed tomography and pulmonary function tests (see Additional file 1).

### Sample preparation

Briefly, the plasma samples of all subjects were collected on an empty stomach in the morning both in the pilot study and the validation study and stored at − 80 °C until metabolic analysis. All samples for the two studies were handled according to the same measurement procedure. Frozen plasma was gently thawed at 4 °C. Then, 100 μL of plasma was added to 300 μL of methanol or acetonitrile, thoroughly mixed on a vortex mixer for 15 s three times, and centrifuged at 12,000 rpm for 5 min. Then, 100 μL of supernatant was pipetted into vials to be analysed on an ultra-high-performance liquid chromatography-mass spectrometry (UHPLC-MS) instrument for UHPLC-MS analysis.

### Metabolomics profiling by UHPLC-MS

**Fig. 1:**
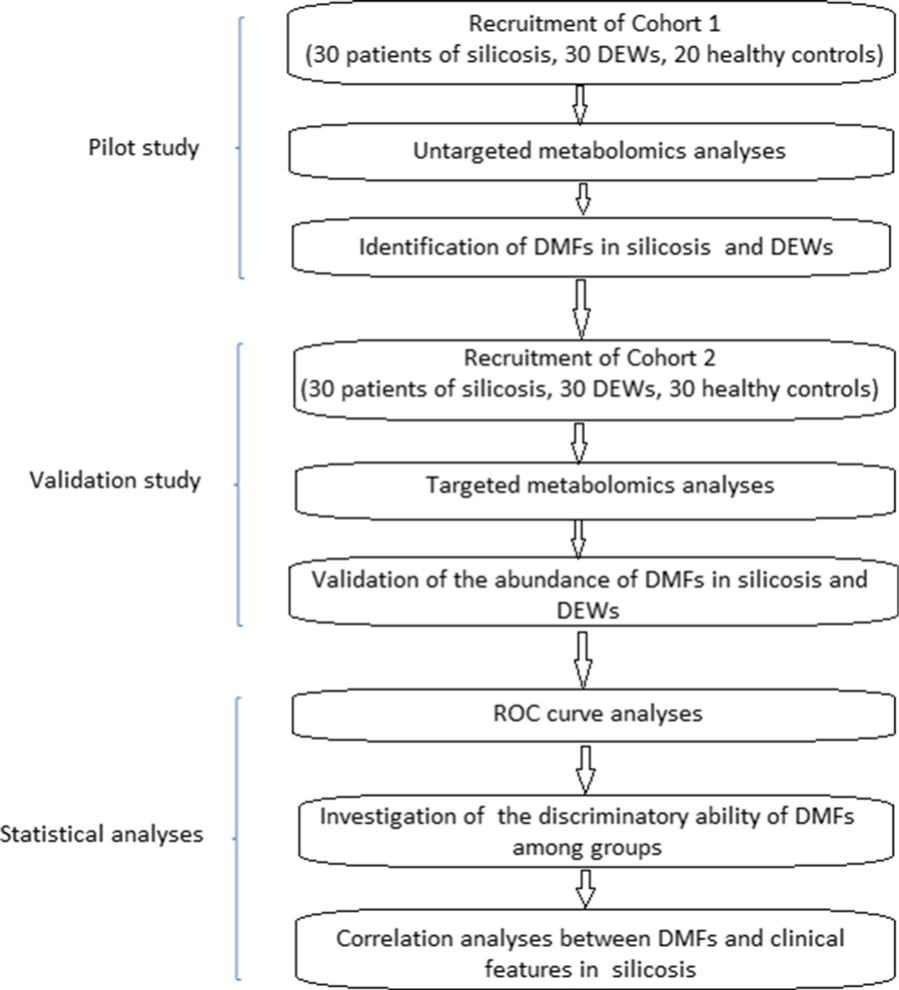
The workflow of our work. The pilot study phase (80 individuals), validation study phase (90 individuals) and statistical analyses were incorporated into our study

The workflow of our work was shown in Fig. 1. The samples were separated by reversed-phase chromatography and hydrophilic chromatography. In this study, the plasma samples were randomly numbered for metabolite extraction and analytical run. All samples were processed and detected continuously within 2 days. Pooled quality control samples were prepared by mixing all of the samples to ensure the data quality of metabolic profiling (see Additional file 1: Figure S1).

For reversed-phase chromatography on a C_18_ column, the plasma samples were melted, chloroform/methanol was added, ultrasonication was conducted, and water was then added to the mixture. After centrifugation, the lower chloroform was concentrated and dried; then, isopropyl alcohol/acetonitrile was added and dissolved by ultrasonication. The solution was centrifuged, and the supernatant was transferred for analysis. Unlike the reversed-phase chromatography, in which hydrophilic chromatographic analysis was conducted on a HILIC column, acetonitrile was added to the melted plasma samples, the mixture fully oscillated, and the upper solution was taken to be tested. The pooled quality-controlled sample was tested at the beginning of the test. Then, every seven samples were tested again to monitor the process to ensure the reliability of the data. A hybrid quadrupole-orbitrap mass spectrometer (Q Exactive, Thermo Fisher Scientific, Beijing, China) equipped with a HESI-II probe was used for mass spectrometry (MS). The liquid quality system was controlled by Xcalibur 2.2 SP1.48 software. Data acquisition and quantitative treatment of targeted metabolites were performed using the same software.

### Statistical analysis

The UHPLC-MS raw data were processed using Progenesis QI (Waters Corporation, Milford, MA, USA), which performed run alignment, peak picking, adduct deconvolution and feature identification against the HMDB database and laboratory-specific reference database. The fragmentation mass spectrum of metabolites was matched to fragmentation mass spectra in library and scored. The results with higher score were accepted as the identification results. Principal component analysis (PCA) and orthogonal partial least-squares-discriminant analysis (OPLS-DA) were performed using Simca (Umetrics Version 14.1).The corresponding parameters of models including R^2^X, R^2^Y, and Q^2^ were obtained and used to ensure the quality of the multivariate models and to avoid the risk of overfitting. *t* tests and variable importance for projection (VIP) statistics were used, and metabolites with significant change were filtered out based on VIP values (VIP > 1) and *t* tests (*P* < 0.05). MetaboAnalyst 4.0 software was used for pathway enrichment analysis. The Kyoto Encyclopedia of Genes and Genomes (KEGG) is a self-sufficient, integrated resource consisting of genomic, chemical and network information.

The validation data were processed using Skyline (MacCoss Lab). Compared with the peak intensity of the standard reference, the concentration of metabolites was calculated and statistical analyses were performed with SPSS version 22.0 (IBM Corp., Armonk, NY, USA). Data are expressed as mean ± standard deviation. Group differences were examined using *t* tests and one-way analysis of variance for continuous variables, chi-square test for count data and the Kruskal–Wallis test for non-normally distributed median values. Correlations between parameters were assessed by Pearson’s correlation coefficient. The levels of the DMFs were further analysed by a receiver operating characteristic (ROC) curve to determine the area under the curve (AUC) and the sensitivity and specificity. A *P* value of < 0.05 was considered statistically significant.

## Results

### Demographics of the study population

The demographics of each group in both the pilot and validation studies were summarized in Table 1. There was no significant difference in sex, age, body mass index or smoking status among the three groups.

**Table 1:** Baseline demographics of the study population

	Pilot phase				Validation phase			
	SIL	DEW	CON	*P* value	SIL	DEW	CON	*P* value
N	30	30	20		30	30	30	
Male:Female	25:5	27:3	14:6	0.148	27:3	27:3	24:6	0.095
Age (years)	59.3 ± 10.1	63.0 ± 4.7	59.5 ± 9.9	0.182	56.2 ± 12.9	60.1 ± 7.9	55.1 ± 11.1	0.176
Smokers: non-smokers	20:10	22:8	11:9	0.405	21:9	20:10	18:12	0.709
Current smokers:ex-smokers	3:17	9:13	4:7	0.166	5:16	7:13	7:11	0.572
BMI (kg/m^2^)	24.6 ± 3.2	23.7 ± 2.3	23.6 ± 2.12	0.345	22.8 ± 3.3	24.4 ± 3.3	23.8 ± 3.2	0.155
Duration of exposure (years)	13.1 ± 7.2	9.3 ± 3.3	NA	0.002	11.8 ± 6.5	10.2 ± 5.0	NA	0.033
FVC (predicted %)	77.6 ± 20.8	82.9 ± 22.3	83.2 ± 15.6	0.047	75.2 ± 23.3	80.4 ± 17.5	82.8 ± 17.4	0.039
FEV_1_ (predicted %)	76.2 ± 19.7	79.6 ± 18.7	80.4 ± 12.1	0.216	77.7 ± 18.0	79.1 ± 19.6	80.7 ± 11.7	0.054
FEV_1_/FVC (%)	81.1 ± 20.4	83.1 ± 24.6	82.8 ± 18.3	0.428	80.5 ± 19.4	82.0 ± 20.0	81.9 ± 14.2	0.366
DL_CO_ (predicted %)	76.7 ± 17.2	80.8 ± 18.4	81.2 ± 10.5	0.105	73.3 ± 15.4	79.4 ± 16.3	80.6 ± 11.1	0.233

*P* values were computed by chi-square test for sex and smoking status, one-way analysis of variance for age, BMI, FVC, FEV_1_, FEV_1_/FVC ratio and DL_CO_. *t* test was used to estimate duration of exposure

*SIL* patients with silicosis, *DEW* dust-exposed workers without silicosis, *CON* healthy control, *BMI* body mass index, *FVC* forced vital capacity, *FEV*_*1*_ forced expired volume in 1 s, *DL*_*CO*_ diffusing capacity of the lung for carbon monoxide, *NA* not available

### PCA and OPLS-DA

**Fig. 2:**
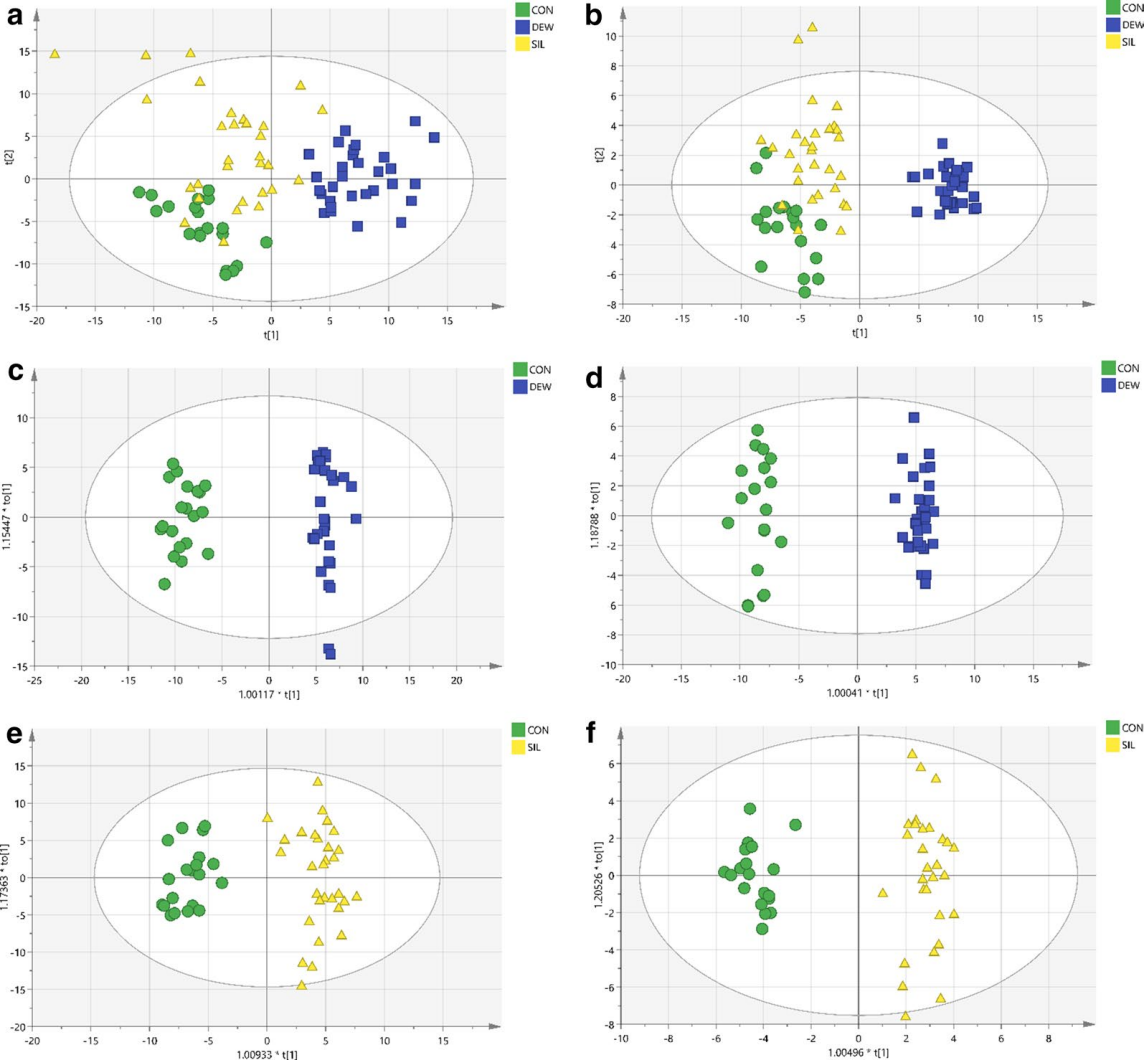
PCA and OPLS-DA of the metabolic profiles of plasma samples in silicosis (SIL), dust-exposed workers (DEW) and healthy control (CON) groups. **a** PCA analysis in C_18_ mode. **b** PCA analysis in HILIC mode. **c** OPLS-DA analysis in C_18_ mode comparing DEW and CON. **d** OPLS-DA analysis in HILIC mode comparing DEW and CON. **e** OPLS-DA analysis in C_18_ mode comparing SIL and CON. **f** OPLS-DA analysis in HILIC mode comparing SIL and CON

In this study, the differentially regulated plasma metabolites in the patients with silicosis, DEWs and healthy controls were searched by nontargeted metabolic profiling using UHPLC-MS, and PCA, using the unsupervised model, was performed to reveal the differences in the metabolic profiles of samples among the groups. The PCA score plot exhibited clear clusters of plasma samples among the silicosis, DEW and control groups both in the C_18_ column (R^2^X = 0.725, Q^2^ = 0.472) and the HILIC column (R^2^X = 0.554, Q^2^ = 0.296), as shown in Fig. 2a, b. For further analysis of the metabolic differences between the controls and DEWs and between the controls and patients with silicosis, OPLS-DA, a supervised method for pattern recognition, was applied. As illustrated by the OPLS-DA score plot, the plasma samples in the DEW group were clearly separated from those in the control group both in the C_18_ column (R^2^Y = 0.973, Q^2^ = 0.952) and the HILIC column (R^2^Y = 0.983, Q^2^ = 0.966) (Fig. 2c, d). Those in the silicosis group were clearly separated from those in the control group both in the C_18_ column (R^2^Y = 0.926, Q^2^ = 0.859) and the HILIC column (R^2^Y = 0.962, Q^2^ = 0.848) (Fig. 2e, f).

### Analysis of the metabolic pathways in the DEWs and patients with silicosis

**Fig. 3:**
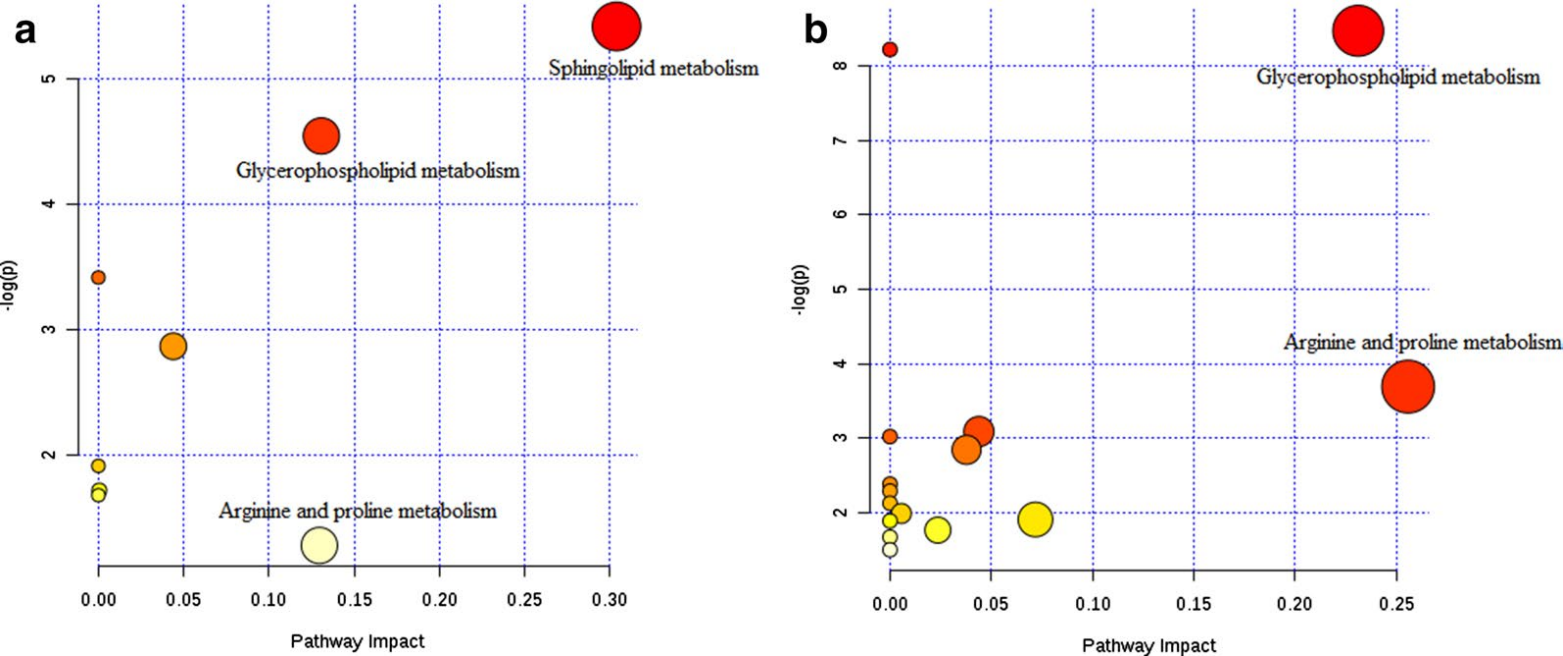
Metabolic pathways in **a** dust-exposed workers without silicosis and **b** the patients with silicosis. Metabolomics view displays matched pathways as circles. The colour and size of each circle is based on the *P* value and pathway impact value, respectively

To identify the metabolic networks and the biological relevance of the identified DMFs in the DEW and silicosis groups, we used MetaboAnalyst 4.0 software and the KEGG database. Sphingolipid metabolism and arginine and proline metabolism were the major metabolic pathways in the DEW and silicosis groups, respectively (Fig. 3).

### Identification of the DMFs in the DEWs and patients with silicosis compared with the controls in the pilot study

Approximately 3336 metabolic features, including 1223 C_18_ positive features, 1069 C_18_ negative features and 1044 HILIC features were found and used for the further statistical analysis. OPLS-DA score plots were used to identify the DMFs for distinguishing the DEWs and patients with silicosis from the controls. According to the cutoff of VIP > 1 and *P* < 0.05 based on OPLS-DA model analysis, 49 DMFs were detected in the plasma of the DEWs; 44 DMFs (21 lipids, 6 amino acids and 17 carnitines) increased and 5 DMFs (4 lipids and 1 amino acid) significantly decreased compared with the controls. Thirty-seven DMFs were detected in the plasma of the patients with silicosis; 24 DMFs (16 lipids, 4 amino acids and 4 carnitines) increased and 13 DMFs (10 lipids and 3 amino acids) significantly decreased compared with the controls.

### Validation of the abundance of the identified DMFs in the validation study

To validate the abundance of the DMFs, we performed targeted metabolomics analysis, and the difference in the abundance of the DMFs among the silicosis, DEW and healthy control groups was analysed in the validation study. The profiles of the DMFs revealed a clear difference between the DEWs and the controls and between the patients with silicosis and the controls in the form of heat maps (Additional file 1: Figure S2, S3).

**Fig. 4:**
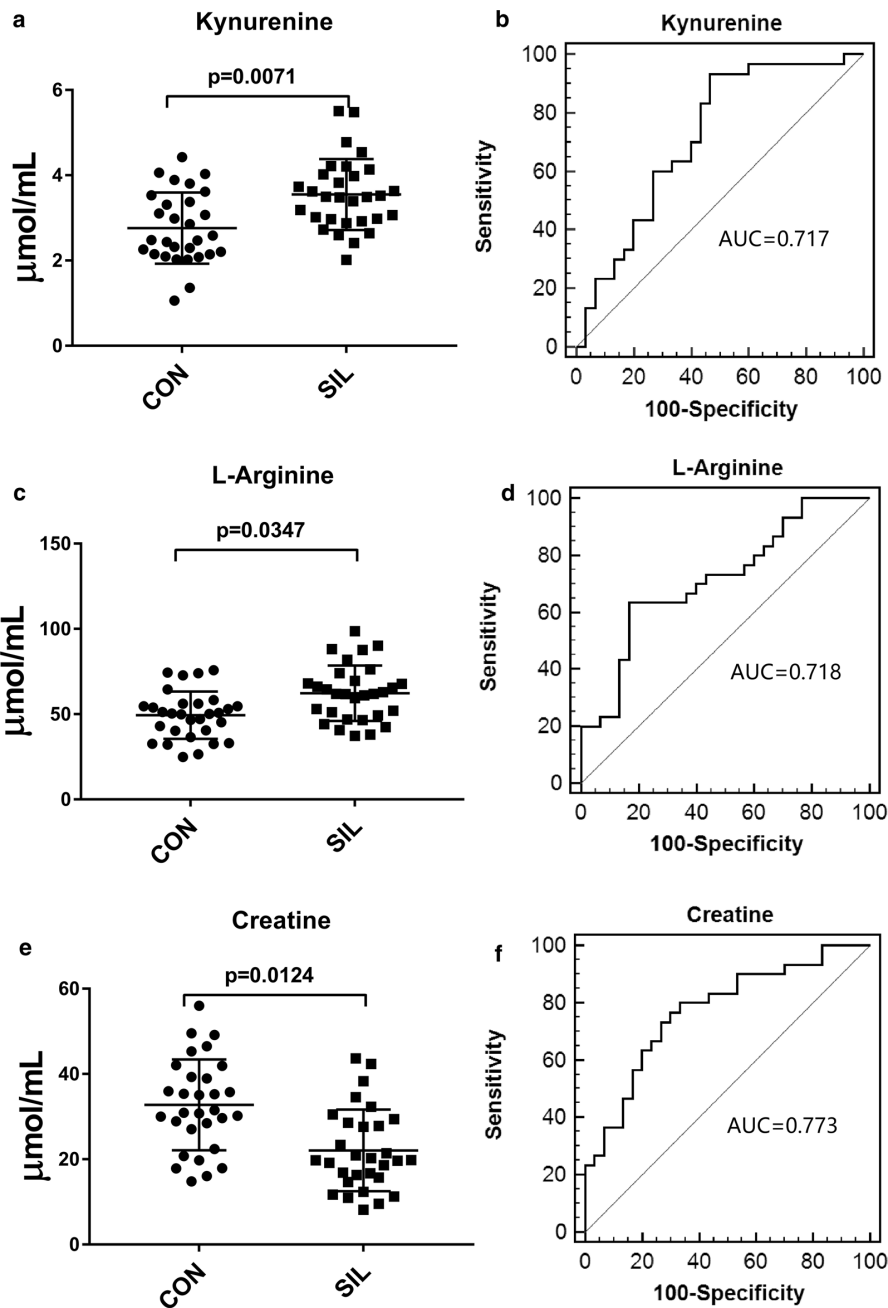
Comparison of the abundance of distinct metabolic features (DMFs) in silicosis (SIL) and healthy control (CON) groups after targeted metabolomics validation and ROC analyses. *P* < 0.05 indicated statistical significance. **a** Abundance of kynurenine. **b** ROC curve of kynurenine. **c** Abundance of l-arginine. **d** ROC curve of l-arginine. **e** Abundance of creatine. **f** ROC curve of creatine

The abundance of 29 identified DMFs in the DEWs and 9 identified DMFs in the patients with silicosis had the same trend in the pilot study and the validation study (Additional file 1: Table S1, S2). To assess the discriminatory ability of all the aforementioned DMFs between the controls and the DEWs and between the controls and the patients with silicosis, we performed ROC curve analyses to calculate the AUC. Twenty of 29 DMFs, mainly carnitines and lipids, had distinct values in the plasma samples of the DEWs compared to those of the controls (Additional file 1: Figure S4). In the plasma samples of the patients with silicosis compared with the controls, 3 amino acid DMFs, kynurenine, l-arginine and creatine, could discriminate between the groups. The abundances and the ROC curves are shown in Fig. 4. These three DMFs were identified and quantified by standard reference materials. MS/MS spectrum from standard reference, pooled plasma sample from control group, silicosis group and all samples were compared, which supported the identification results of the metabolites (Additional file 1: Figure S5-S7).

### Correlations between DMFs and the severity of silicosis

To explore the correlation between the three DMFs and the severity of silicosis, we classified all patients with silicosis into three stages according to the diagnostic criteria of pneumoconiosis based on the 2011 ILO classification. The abundance of kynurenine was higher in Stage III silicosis than in Stage I or Stage II silicosis. However, those of creatine and l-arginine were not significantly different among the various stages of silicosis (Table 2).

**Table 2:** Plasma concentrations of kynurenine, l-arginine and creatine in the patients with various stages of silicosis

	Stage I	Stage II	Stage III	*P* value
N	9	8	13	
Kynurenine (μmol/mL)	3.1 ± 0.4	3.1 ± 0.7	4.1 ± 0.8*^▲^	0.002
l-arginine (μmol/mL)	71.4 ± 14.0	58.7 ± 19.2	58.3 ± 14.4	0.137
Creatine (μmol/mL)	24.8 ± 8.9	22.8 ± 10.4	19.8 ± 9.7	0.480

Data was presented as mean ±SD

*Compared with Stage I, *P* value < 0.05; ^▲^Compared with Stage II, *P* value < 0.05

**Fig. 5:**
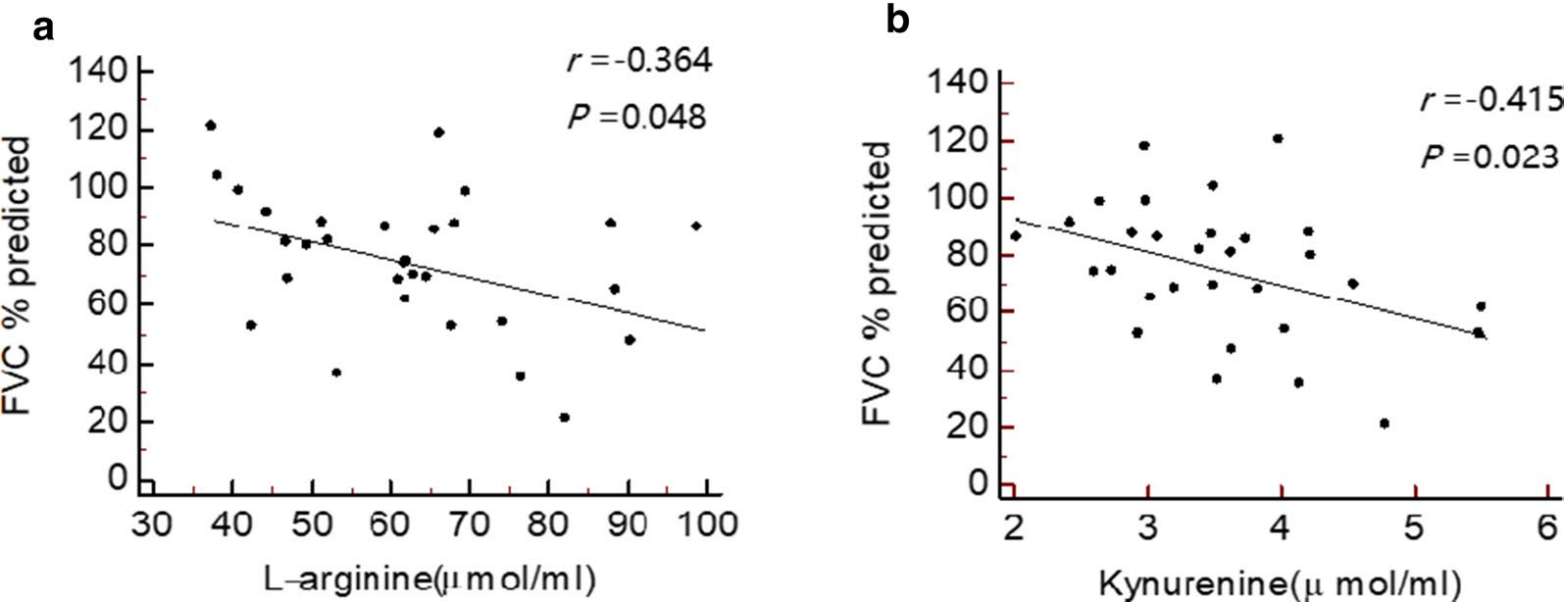
Correlations between l-arginine and kynurenine and the predicted percentage of forced vital capacity (FVC% predicted) in silicosis. **a** l-arginine and FVC% predicted. **b** Kynurenine and FVC% predicted

Pulmonary function was assessed for each patient with silicosis in the validation study. As shown in Fig. 5, the abundances of l-arginine and kynurenine were negatively correlated with the predicted percentage of forced vital capacity (FVC% predicted) (*P* < 0.05) but not correlated with other lung function values in silicosis.

## Discussion

Silicosis is a well-known fibrotic lung disease caused by prolonged inhalation of crystalline silica. In order to explore the metabolic mechanism of silicosis, we designed this study to compare the metabolic features of the patients of silicosis and DEWs in metabolomics studies. Twenty-nine DMFs were identified in the plasma of the DEWs in the pilot and validation study. Sphingolipid metabolism was the major metabolic pathway in the DEWs. It showed that the metabolic mechanism was decidedly altered once dusts entered into the respiratory tracts in the dust exposure environment. However in the patients with silicosis, 9 DMFs were detected to be consistent in the pilot and validation study. Arginine and proline metabolism was the major metabolic pathway in silicosis.

In the present study, 20 DMFs, mainly lipids and carnitines, could distinguish the DEWs from the healthy controls. Lipids are essential nutrients in humans and are the main components of cell membranes and cellular energy storage. Lipids are related to signal transduction, enzyme activation, growth factors and antioxidants, signal recognition and immunity [21–23]. Recently, the role of lipids has attracted increased attention in lungs and respiratory diseases, including cystic fibrosis, asthma and chronic obstructive pulmonary disease, which are all associated with abnormal metabolisms [24–26]. Researchers have revealed vital information regarding lipid metabolism in IPF patients, and more importantly, a few potentially promising biomarkers were first identified and may have a predictive role in monitoring and diagnosing IPF [18, 27, 28]. Lysophosphatidylcholine (LysoPC), an intermediate metabolite of sphingolipids, was shown to be a potential biomarker in the serum of patients with IPF by pilot and further validation studies using UHPLC-MS [29]. Carnitine is related to the metabolism of fat into energy in the body and can promote the transport and oxidation of fatty acids and the utilization of carbohydrates and amino acids, improve body tolerance, prevent lactic acid accumulation, and delay ageing and is involved in antioxidant activities [30]. Carnitine was significantly decreased in the lung tissue and reduced mitochondrial beta-oxidation in IPF [17]. In the early phase of silicosis, the enhancement of macrophage phagocytosis, apoptosis, oxidative stress and inflammation is caused by inhaled crystalline silica [31–33] and is closely related to the abnormal metabolism of lipids and carnitines. The data suggested that sphingolipid metabolism was the major metabolic pathway in the DEWs and that LysoPC and lysophosphatidylethanolamine (LysoPE), the intermediate metabolites of sphingolipid metabolism, were upregulated.

In the present study, the distinct metabolic features in the plasma of the patients of silicosis compared with that of DEWs were identified to be different. Five lipid DMFs were still metabolism increased in silicosis, such as phosphatidylcholine and LysoPE. Three amino acid DMFs, l-arginine, kynurenine and creatine, could distinguish patients with silicosis from the healthy controls with ROC curve analysis. Amino acids are the basic components of proteins and the raw materials for protein synthesis, and they participate in the complex metabolic process of the body. Increased levels of some amino acids, including creatine, putrescine, spermidine, 4-hydroxyproline and proline-hydroxyproline dipeptide, were found in the fibrotic lung tissue of patients with IPF compared with normal lung tissue [17]. Collagen fibrils are the most abundant protein in the ECM, and excess collagen deposition in the ECM is associated with the key pathogenic mechanism of IPF [34]. Ornithine can also be converted to proline and hydroxyproline for collagen formation in fibrosis [18]. Silicosis has similar changes in amino acid metabolism as IPF, which may be related to the fibrotic process [19]. In the late pathogenesis of silicosis, the formation of silicotic nodules is the pathological manifestation of the production of collagen fibres and pulmonary interstitial fibrosis mediated by amino acid metabolism, which is consistent with the finding in the present study that arginine and proline metabolism was the major metabolic pathway in silicosis.

A growing number of studies have suggested that arginine methylation and asymmetric dimethylarginine metabolism may be associated with the progression of IPF [35]. One animal experiment featuring lung fibrosis showed that the direct infusion of asymmetric dimethylarginine resulted in elevated collagen deposition in mouse lungs and enhanced arginase activity [36]. l-arginine can produce l-ornithine under the action of arginase, and then, through ornithine aminotransferase, l-ornithine converts into proline, which is the main component of collagen [37]. Our data has shown that the plasma level of l-arginine in silicosis was significantly higher than that in the controls and was related to the decline of pulmonary function. Kynurenine is an intermediate metabolite of tryptophan, an essential amino acid. Kynurenine has immune regulatory functions and can regulate vascular tone, which might be relevant in pulmonary hypertension [38]. Pulmonary hypertension is related to pulmonary artery stenosis resulting from mechanical compression by the lesions of central type progressive massive fibrosis in silicosis. We found that compared with that of the controls, the level of kynurenine significantly increased in silicosis. Moreover, the level of kynurenine was higher in Stage III silicosis than in Stage I or Stage II silicosis and was negatively associated with FVC% predicted. This finding may partially explain the overexpression of kynurenine in severe silicosis.

Some limitations of the present study should be mentioned. First, potential enrolment bias existed in the present study and may affect the validity of the results. The study population from a single medical centre may not be fully representative of all patients with silicosis. In addition, more males were enrolled than females because they were at risk of silica dust exposure through engagement in manual labour, such as excavation and digging, polishing and buffing. Second, other lung diseases such as chronic obstructive pulmonary disease and some exogenous factors such as smoking, alcohol and medication may also lead to metabolic changes [39, 40]. These were not included in this study. The possible effects of these factors were warranted to be assessed in further study. Third, although the plasma metabolic features of silicosis are accessible and noninvasive, they may not fully represent the metabolic process of the lungs. Further research is warranted to explore the distinct metabolism of sputum as well as bronchoalveolar lavage in patients with silicosis. Finally, given the cross-sectional design, the study did not have the power to explore the dynamic metabolites for disease progression and survival, which are clinically significant.

## Conclusions

The present study provided important information regarding sphingolipid metabolism and arginine and proline metabolism in DEWs and patients with silicosis, respectively. The distinct metabolic features in the plasma of the patients of silicosis compared with that of DEWs were found to be different. In the validation metabolomics study, our results showed that l-arginine and kynurenine were correlated with the severity of silicosis, and may have a predictive role in monitoring this disease. Further study is warranted to explore the metabolic mechanisms and the possibility of intervention in or prevention of silicosis.

## Supplementary Information

**Additional file 1.** Additional file contains supplemental methods and supplemental Figures and Tables as referenced in the manuscript.

## Data Availability

The datasets used and/or analysed during the current study are available from the corresponding author on reasonable request.

## Abbreviations

AUC: Area under the curve
DEW: Dust-exposed worker
DMFs: Distinct metabolic features
ECM: Extracellular matrix
FVC: Forced vital capacity
ILO: International Labour Organization
IPF: Idiopathic pulmonary fibrosis
KEGG: Kyoto Encyclopedia of Genes and Genomes
LysoPC: Lysophosphatidylcholine
LysoPE: Lysophosphatidylethanolamine
MS: Mass spectrometry
OPLS-DA: Orthogonal partial least-squares-discriminant analysis
PCA: Principal component analysis
QC: Quality control
ROC: Receiver operating characteristic
UHPLC-MS: Ultra-high performance liquid chromatography-mass spectrometry
VIP: Variable importance for projection
