# Supplementary material for: Distinct metabolic features in the plasma of patients with silicosis and dust-exposed workers in China: a case–control study
Source: BMC Pulm Med. 2021 Mar 17;21:91. doi: 10.1186/s12890-021-01462-1 (PMC7971960; doi:10.1186/s12890-021-01462-1)
Supplement: Supplementary file 1 — Additional file 1. Additional file contains supplemental methods and supplemental Figures and Tables as referenced in the manuscript. [file 12890_2021_1462_MOESM1_ESM.docx]

**Distinct metabolic features in the plasma of patients with silicosis and dust-exposed workers in China：a case-control study**

Changjiang Xue, Na Wu, Yali Fan, Jing Ma, Qiao Ye

Department of Occupational Medicine and Toxicology, Clinical Centre for Interstitial Lung Diseases, Beijing Chao-Yang Hospital, Capital Medical University, Beijing, 100020, China

**Corresponding author**

Qiao Ye, Department of Occupational Medicine and Toxicology, Clinical Centre for Interstitial Lung Diseases, Beijing Chao-Yang Hospital, Capital Medical University;

Add.: No. 8 Workers’ Stadium South Road, Chao-Yang District, Beijing, China;

Tel: +86-010-85231799;

E-mail: [yeqiao_chaoyang@sina.com](mailto:yeqiao_chaoyang@sina.com)

**Supplemental methods**

**Classification of silicosis by chest radiograph**

Conventional chest radiographs were performed in each patient with silicosis and independently evaluated by two occupational medicine experts. Silicosis was classified into three stages according to the International Labour Organization classification system. Briefly, each lung field was divided into three zones (upper, middle, lower) on the posterior chest radiographs. When the highest density of small opacities was ≥1/0, the distribution affected two or more zones and pleural plaques were apparent, the patients were diagnosed as Stage I. When the highest density of small opacities was ≥2/1 and the distribution affected more than four zones, or the highest density of small opacities was ≥3/2 and the distribution affected four or more zones, the patients were diagnosed as Stage II. When the highest density of small opacities was ≥3/2 and the distribution affected four or more zones with aggregation of small or large opacities, or the diameter of the largest opacity was ≥20 × 10 mm, the patients were diagnosed as Stage III. The interobserver correlation was good, and the κ value was 0.841.

**High-resolution computed tomography (HRCT)**

HRCT was acquired on a 64-slice single-source computed tomography (CT) system with 0.625–mm sections, a 1–sec scan time and a 10-mm interval in the apex–base scans, with the inclusion of both lungs in the field of view. Large opacity was defined as an opacity having the largest diameter (at the mediastinal window setting) >1 cm. Two experts independently assessed the presence of large opacity on HRCT, according to the International Classification of HRCT for Occupational and Environmental Respiratory Diseases (ICOERD) with good interobserver correlation (0.762).

**Pulmonary function test**

Pulmonary function tests were carried out by certified technicians according to hospital guidelines, which met the quality control standards established jointly by the American Thoracic Society and European Respiratory Society. Parametersused for analysis of the flow–volume curve were the forced vital capacity (FVC), forced expired volume in the first second (FEV_1_), and FEV_1_/FVC ratio. Each participant also underwent evaluation of their total lung capacity and diffusing capacity of the lung for carbonmonoxide (DL_CO_SB) (single-breath method, with the values corrected for the present hemoglobin concentration).The results are expressed as percentages of predicted values on the basis of age, height, and sex using equations established by the European Respiratory Society. The forced expiratory maneuvers were repeated until three sequential measurements were obtained. The indices were obtained from the best curve, which was associated with the highest value of FEV_1_ plus FVC.

**Quality control evaluation**

In this study, 5 system blank samples and 5 quality control (QC) samples were added before experimental sample analysis list. System blank samples were used to monitor impurities present in the analysis system. 5 QC samples were used to equilibrate the system to achieve reproducible conditions. Pooling equal volume mixture of all plasma samples, QC samples were prepared and processed using the same method with other plasma samples. QC samples were injected eight samples a time in order to assess the data repeatability. Unsupervised principal component analysis (PCA) was used to analyze the data of QC samples and other experimental samples. The aggregation of QC samples in PCA diagram can reflect the stability of sample detection. The PCA plots of the metabolic profiles of plasma samples in different modes were shown in Supplemental Figure 1.

**Supplemental figures**


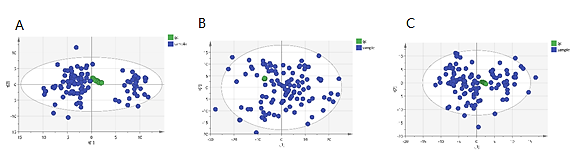


**Supplemental Figure 1** The PCA plots of the metabolic profiles of plasma samples in (A) HILIC mode, (B) C_18_ positive mode and (C) C_18_ negative mode. The green dots represent the quantity control (QC) samples and the blue dots represent the experimental samples.


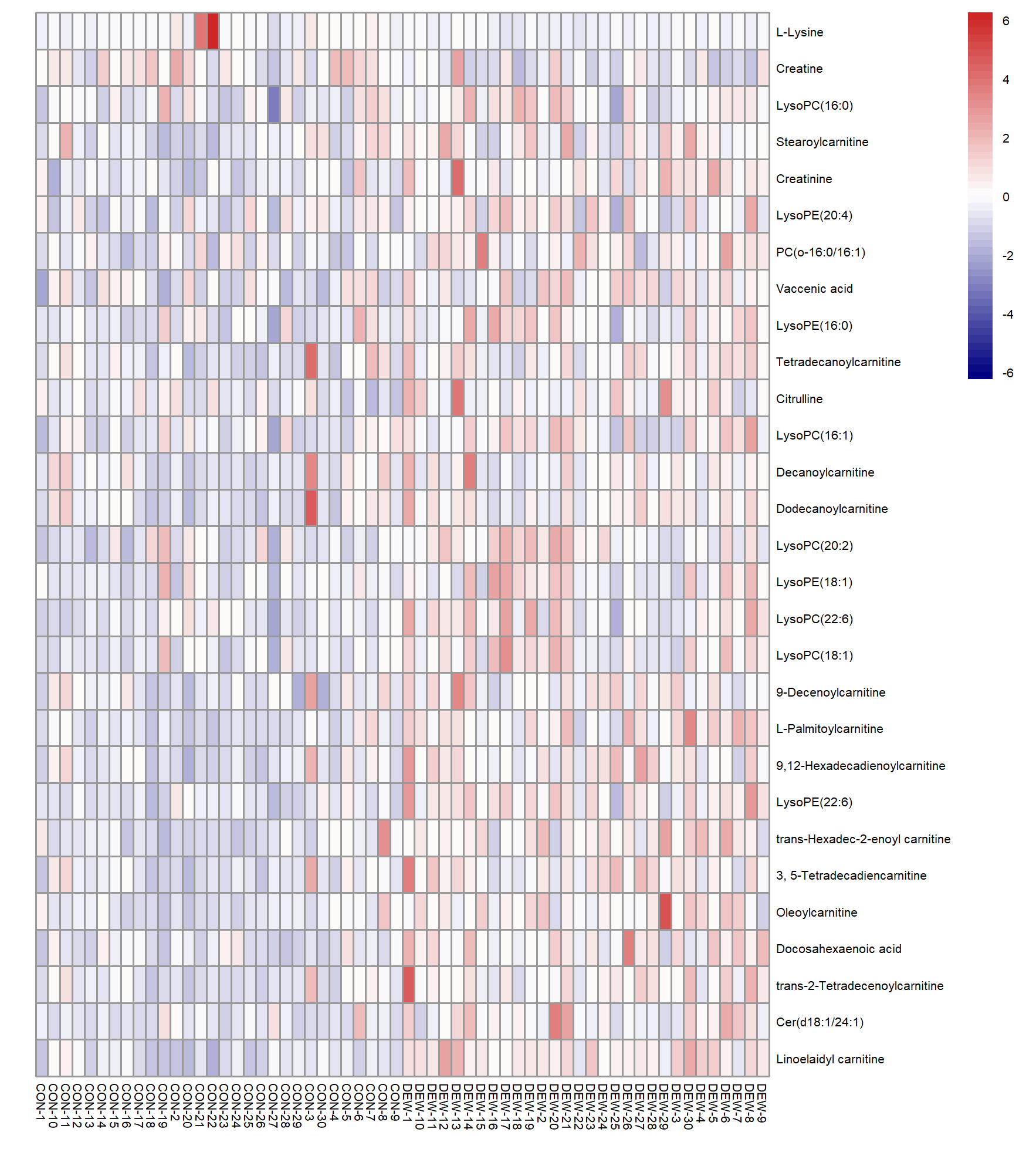


**Supplemental Figure 2** Heat maps representing the abundance profile of the distinct metabolic features between dust-exposed workers (DEW) without silicosis and healthy controls (CON). Identity of the metabolic features was shown on the right side, individual samples in rows, respectively. Cells colored in red represent up-regulated, and cells colored in blue represent down-regulated abundances. The analysis was done with the MetaboAnalyst online platform.


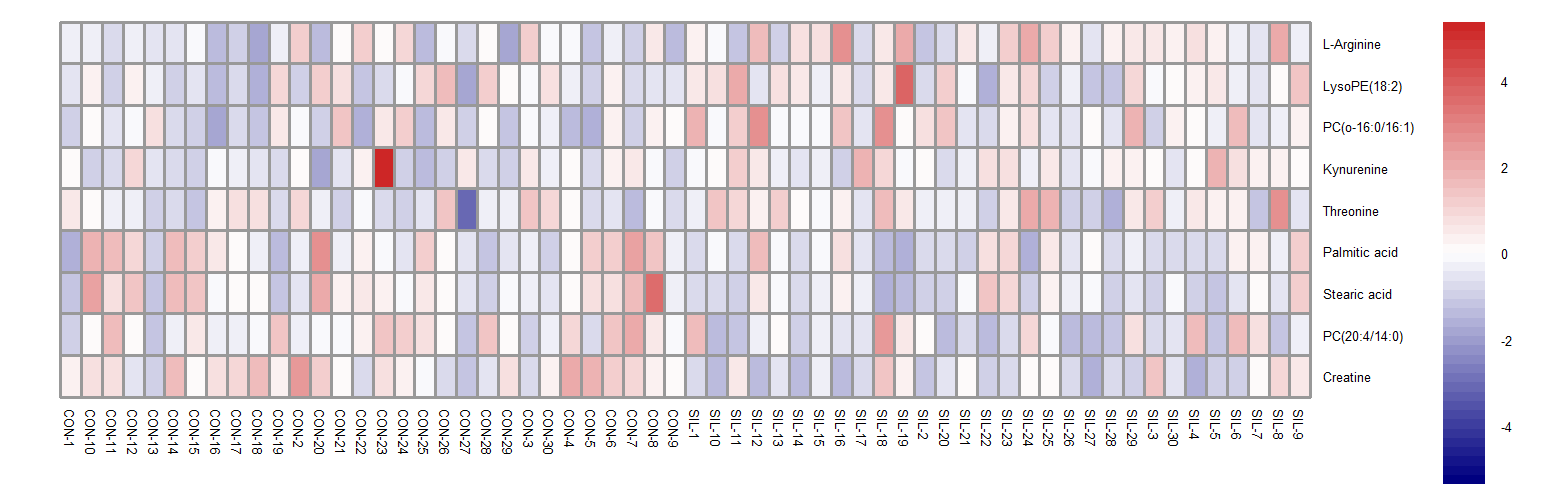


**Supplemental Figure 3** Heat maps representing the abundance profile of the distinct metabolic features between the patients with silicosis (SIL) and healthy controls (CON). Identity of the metabolic features was shown on the right side, individual samples in rows, respectively. Cells colored in red represent up-regulated, and cells colored in blue represent down-regulated abundances. The analysis was done with the MetaboAnalyst online platform.


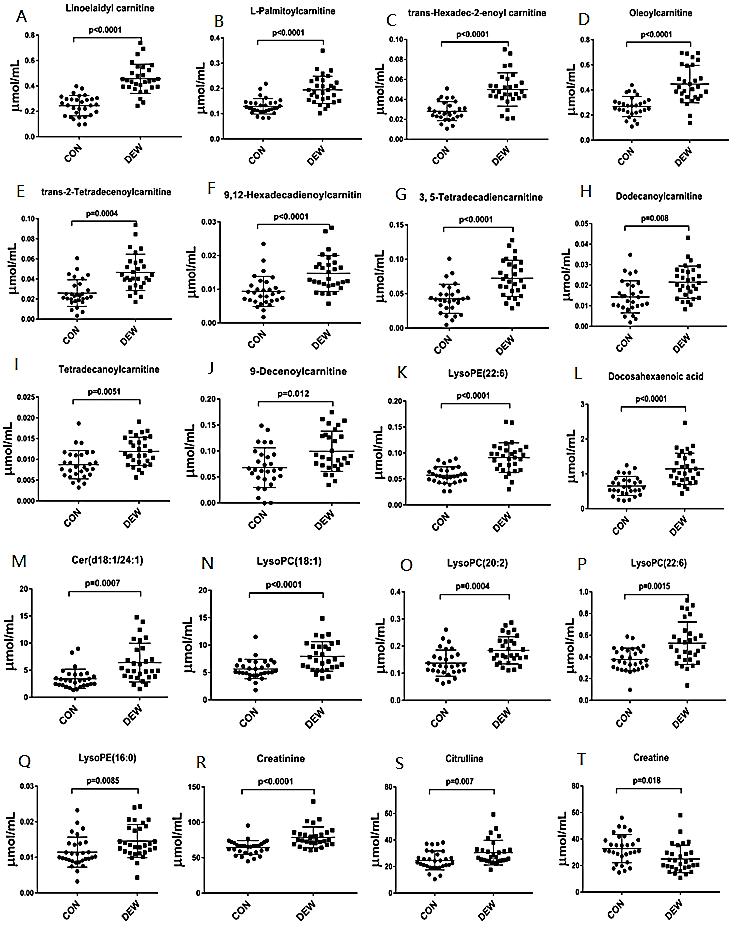


**Supplemental Figure 4** Comparison of the abundance of the distinct metabolic features in dust exposure workers (DEWs) without silicosis and healthy control (CON) groups after targeted metabolomics validation and ROC analyses. *P*< 0.05 indicated statistical significance.

**
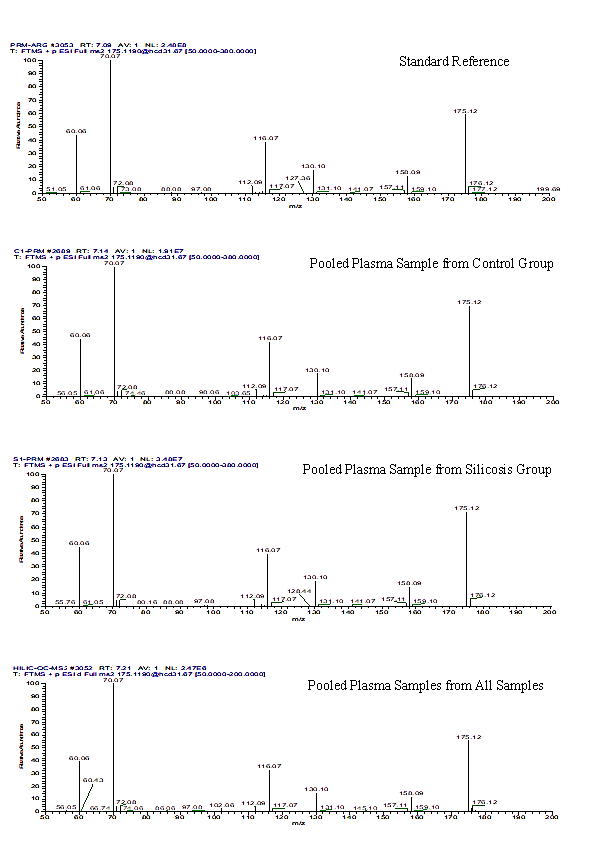
**

**Supplemental Figure 5** Mass spectrometry (MS)/MS spectrum of L-arginine in different samples (m/z 175.1190, RT 7.14 min).

**
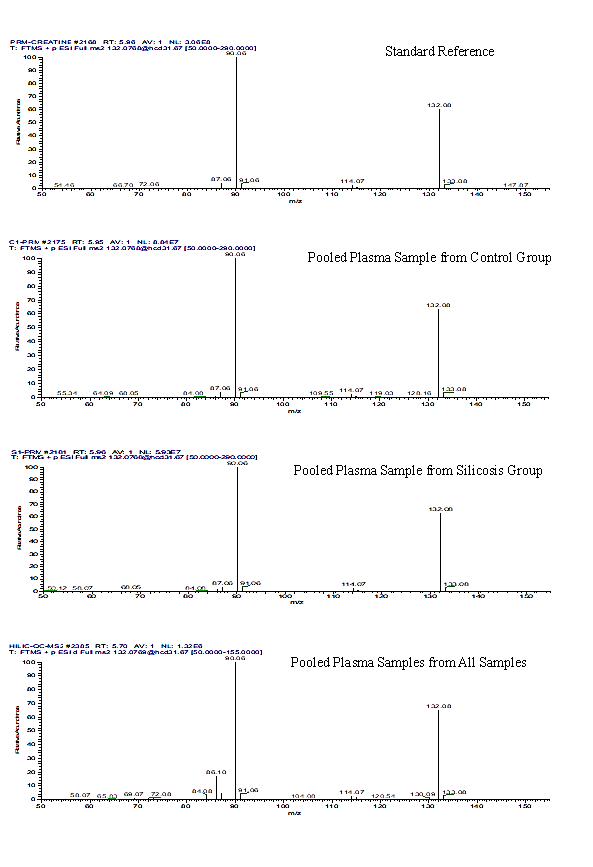
**

**Supplemental Figure 6** Mass spectrometry (MS)/MS spectrum of creatine in different samples (m/z 132.0768, RT 5.96 min).


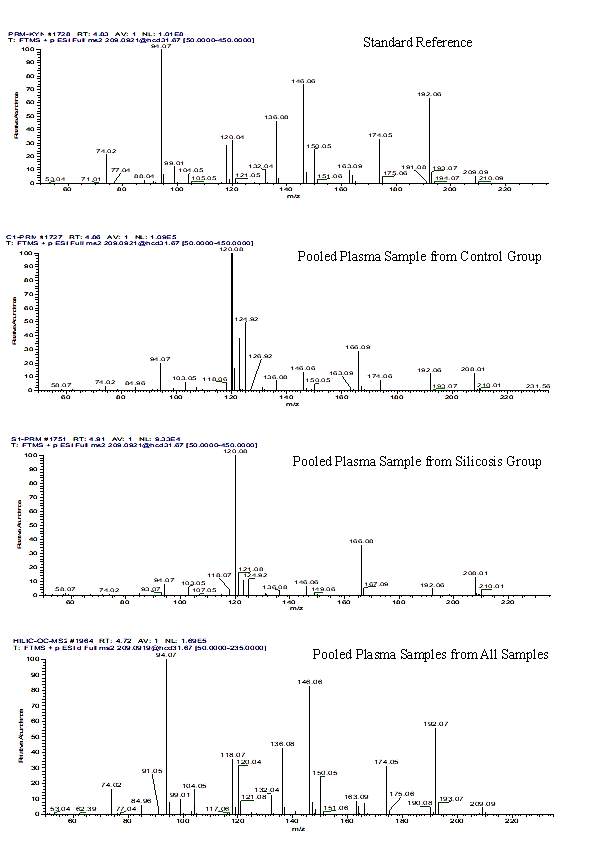


**Supplemental Figure 7** Mass spectrometry (MS)/MS spectrum of kynurenine in different samples (m/z 209.0921, RT 5.96 min).

**Supplemental tables**

**Supplemental Table 1** List of assigned statistically significantly DMFs after comparison of DEWs to healthy controls

| No | Compund | Formula | m/z | Fold change | VIP | *P* value |
| --- | --- | --- | --- | --- | --- | --- |
| 1 | Creatinine | C_4_H_7_N_3_O | 114.0664 | 1.33 | 1.35578 | 9.87E-09 |
| 2 | Creatine | C_4_H_9_N_3_O_2_ | 132.0768 | 0.58 | 1.00349 | 0.000162 |
| 3 | L-Lysine | C_6_H_14_N_2_O_2_ | 147.1127 | 1.31 | 1.07589 | 0.000108 |
| 4 | Citrulline | C_6_H_13_N_3_O_3_ | 176.1029 | 1.29 | 1.47938 | 0.001383 |
| 5 | Vaccenic acid | C_18_H_34_O_2_ | 281.2479 | 1.82 | 1.60362 | 0.000591 |
| 6 | 9-Decenoylcarnitine | C_17_H_31_NO_4_ | 314.2322 | 1.68 | 1.03997 | 0.000549 |
| 7 | Decanoylcarnitine | C_17_H_33_NO_4_ | 316.2479 | 1.59 | 1.00507 | 3.76E-06 |
| 8 | Docosahexaenoic acid | C_22_H_32_O_2_ | 327.2325 | 2.28 | 1.5569 | 0.000138 |
| 9 | Dodecanoylcarnitine | C_19_H_37_NO_4_ | 344.2792 | 2.57 | 1.1982 | 2.56E-06 |
| 10 | 3, 5-Tetradecadiencarnitine | C_21_H_37_NO_4_ | 368.2791 | 2.32 | 1.14665 | 2.56E-06 |
| 11 | trans-2-Tetradecenoylcarnitine | C_21_H_39_NO_4_ | 370.2948 | 2.98 | 1.16016 | 3.79E-07 |
| 12 | Tetradecanoylcarnitine | C_21_H_41_NO_4_ | 372.3106 | 2.28 | 1.35387 | 4.88E-08 |
| 13 | 9,12-Hexadecadienoylcarnitine | C_23_H_41_NO_4_ | 396.3104 | 2.37 | 1.20337 | 8.61E-07 |
| 14 | trans-Hexadec-2-enoyl carnitine | C_23_H_43_NO_4_ | 398.3262 | 2.51 | 1.20725 | 1.11E-08 |
| 15 | L-Palmitoylcarnitine | C_23_H_45_NO_4_ | 400.3417 | 1.70 | 1.22433 | 8.48E-08 |
| 16 | Linoelaidylcarnitine | C_25_H_45_NO_4_ | 424.3415 | 2.22 | 1.11106 | 9.46E-08 |
| 17 | Oleoylcarnitine | C_25_H_47_NO_4_ | 426.3574 | 2.66 | 1.71806 | 4.32E-09 |
| 18 | Stearoylcarnitine | C_25_H_49_NO_4_ | 428.3738 | 1.60 | 1.07869 | 5.00E-06 |
| 19 | LysoPE(16:0) | C_21_H_44_NO_7_P | 454.2924 | 1.32 | 1.20662 | 0.036689 |
| 20 | LysoPE(18:1) | C_23_H_46_NO_7_P | 480.3085 | 1.90 | 1.37356 | 2.36E-05 |
| 21 | LysoPC(16:1) | C_24_H_48_NO_7_P | 494.3241 | 1.49 | 1.03094 | 0.001697 |
| 22 | LysoPC(16:0) | C_24_H_50_NO_7_P | 496.3391 | 1.34 | 1.73954 | 7.02E-05 |
| 23 | LysoPE(22:6) | C_25_H_44_NO_7_P | 502.2925 | 2.03 | 1.06968 | 1.05E-05 |
| 24 | LysoPC(18:1) | C_26_H_52_NO_7_P | 522.3552 | 1.81 | 1.12145 | 2.11E-06 |
| 25 | LysoPE(20:4) | C_27_H_44_NO_7_P | 526.292 | 1.77 | 1.02334 | 0.001048 |
| 26 | LysoPC(20:2) | C_28_H_54_NO_7_P | 548.3716 | 1.81 | 1.17706 | 8.61E-07 |
| 27 | LysoPC(22:6) | C_30_H_50_NO_7_P | 568.3393 | 2.03 | 1.12556 | 4.33E-05 |
| 28 | Cer(d18:1/24:1) | C_42_H_81_NO_3_ | 648.6283 | 1.97 | 1.27691 | 4.55E-06 |
| 29 | PC(o-16:0/16:1) | C_40_H_80_NO_7_P | 718.5736 | 0.84 | 1.06874 | 0.042377 |

The variable importance for projection (VIP) statistics and t-test were used applied to select significant variables leading to group separation. VIP values of > 1.0 and *P* values of < 0.05 were considered statistically significant. Abbreviations: LysoPE, lysophosphatidyl ethanolamine. LysoPC, lysophosphatidylcholine. PC, phosphatidylcholine. Cer, ceramide

**Supplemental Table 2** List of assigned statistically significantly DMFs after comparison of the patients with silicosis to healthy controls

| No | Compund | Formula | m/z | Fold change | VIP | *P* value |
| --- | --- | --- | --- | --- | --- | --- |
| 1 | Threonine | C_4_H_9_NO_3_ | 120.0655 | 0.70 | 1.79124 | 4.79E-05 |
| 2 | Creatine | C_4_H_9_N_3_O_2_ | 132.0768 | 0.48 | 1.65789 | 3.07E-05 |
| 3 | L-Arginine | C_6_H_14_N_4_O_2_ | 175.1189 | 1.52 | 1.52316 | 0.000231 |
| 4 | Kynurenine | C_10_H_12_N_2_O_3_ | 209.0921 | 1.66 | 1.64859 | 1.05E-05 |
| 5 | Palmitic acid | C_16_H_32_O_2_ | 255.2322 | 1.33 | 2.42667 | 2.52E-05 |
| 6 | Stearic acid | C_18_H_36_O_2_ | 283.2635 | 1.51 | 1.794 | 2.15E-12 |
| 7 | LysoPE(18:2) | C_23_H_44_NO_7_P | 478.2924 | 2.33 | 2.47867 | 0.000474 |
| 8 | PC(o-16:0/16:1) | C_40_H_80_NO_7_P | 718.5736 | 0.72 | 1.44861 | 0.001719 |
| 9 | PC(20:4/14:0) | C_42_H_76_NO_8_P | 754.5375 | 0.667 | 1.44874 | 0.011574 |

The variable importance for projection (VIP) statistics and t-test were used applied to select significant variables leading to group separation. VIP values of > 1.0 and *P* values of < 0.05 were considered statistically significant. Abbreviations: LysoPE, lysophosphatidyl ethanolamine. PC, phosphatidylcholine.
